# Apical anatomy of primary molar root canals: a micro-Ct study

**DOI:** 10.1186/s12903-026-09231-4

**Published:** 2026-07-15

**Authors:** Ashraf Yassin Alhosainy, Jeffrey A Dean

**Affiliations:** 1 Department of Pediatric Dentistry and Dental Public Health, Faculty of Dentistry, Mansoura University, El Gomhouria St., Mansoura, 35516 Egypt; 2 McDonald Professor Emeritus Pediatric Dentistry, Orthodontics and Dentofacial Orthopedics, Indiana University and Riley Children’s Hospital, Indianapolis, Indiana 46202 United States

**Keywords:** Primary molars, Apical anatomy, Micro-computed tomography, Root canal morphology, Egyptian population

## Abstract

**Objective:**

To investigate, using micro–computed tomography (micro-CT), the apical anatomy of mandibular second primary molar roots in Egyptian children, with emphasis on the spatial relationship between the apical foramen and the anatomic apex, and the morphology and dimensions of the root canal at the apical foramen level.

**Materials and methods:**

Fifty extracted human mandibular second primary molars (150 canals: 50 distal, 50 mesiobuccal, 50 mesiolingual) with complete root length and minimal resorption were scanned using high-resolution micro-CT (15.24 μm voxel size). The distance between the apical foramen and the apex, canal major diameter, and roundness at the foramen level were measured using CTAn software. Descriptive statistics were calculated, and exploratory canal-type comparisons were performed using one-way ANOVA followed by Tukey post-hoc tests. (α = 0.05).

**Results:**

The apical foramen did not coincide with the anatomic apex in any canal. The mean apex-to-foramen distance was 0.57 ± 0.44 mm. Mean apical canal diameter was 0.63 ± 0.24 mm and mean roundness was 0.48 ± 0.17, indicating predominantly oval canal shapes. The overall comparison suggested variation in apex-to-foramen distance among canal types (*p* = 0.040), but post-hoc comparisons did not confirm statistically significant pairwise differences between individual canal types. Distal canals exhibited significantly larger diameters than both mesial canals (*p* < 0.05), whereas the difference between mesiobuccal and mesiolingual canals was not significant. Roundness did not differ significantly among canal types (*p* = 0.079).

**Conclusions:**

In Egyptian children, the apical foramen of mandibular second primary molar is consistently offset from the anatomic apex. The distal canals demonstrated the largest major canal diameter at the apical foramen level.

**Clinical relevance:**

Because the apical foramen rarely coincides with the apex and complete mechanical enlargement to the true apical diameter is often clinically unrealistic, pediatric endodontic treatment should prioritize accurate working-length determination (e.g., electronic apex locators) and combine conservative shaping with effective chemical disinfection to optimize apical cleaning while preserving root integrity.

## Introduction

Early childhood caries remains a prevalent oral health problem among children worldwide [1]. Primary teeth should be retained until physiologic exfoliation because they serve as natural space maintainers and contribute to mastication, alveolar bone development, phonation, esthetics, and normal occlusal development [2]. Pulpectomy and root canal treatment remain the preferred treatment options for carious primary teeth with irreversible pulp inflammation or necrosis [3].

Pediatric endodontics has gained increasing clinical attention in recent years. The introduction of rotary nickel–titanium (NiTi) systems has encouraged more clinicians to perform pulpectomy in primary teeth, as these systems improve shaping efficiency, reduce operator fatigue, and shorten chairside time, potentially improving obturation quality [4]. However, successful endodontic treatment requires thorough knowledge of root and canal anatomy, particularly in primary molars, which frequently demonstrate complex and highly variable internal canal configurations [5].

Root canal morphology in primary teeth has been investigated using several techniques, including histology [6], demineralization and dye perfusion [7], scanning electron microscopy [8], and conventional radiography. More recently, three-dimensional imaging modalities such as cone-beam computed tomography (CBCT) and micro-computed tomography (micro-CT) have enabled more accurate visualization of canal anatomy [9, 10]. Micro-CT is a non-invasive and reproducible technique that provides detailed three-dimensional qualitative and quantitative data at high resolution [11]. However, it is limited to in vitro investigations.

The apical region represents a critical and challenging part of the root canal system, as it is the most inaccessible area for instrumentation and irrigation and may present anatomical variations such as multiple foramina and apical deltas [5]. Its clinical relevance is mainly related to its proximity to the periapical tissues and developing permanent successors. Moreover, the optimal apical extent of instrumentation and obturation remains an important determinant of treatment success. Despite this, the apical anatomy of primary teeth has received limited attention.

Therefore, this in vitro study aimed to investigate, using micro-CT, the apical anatomy of mandibular second primary molar roots of Egyptian children, with emphasis on:

1. The spatial relationship between the apical foramen and the anatomic apex, and
2. The morphology and dimensions of root canal at the apical foramen level.

## Methods

### Ethical considerations

The study protocol was approved by the Research Ethics Committee of the Faculty of Dentistry, Mansoura University (Approval No. R.25.11.84). Written informed consent was obtained from parents or legal guardians for the use of extracted primary molars in research. This manuscript was prepared in accordance with the Preferred Reporting Items for Laboratory studies in Endodontology (PRILE) guidelines [12].

### Study design

This in vitro study was performed on extracted human mandibular second primary molars collected from the outpatient pediatric dental clinic, Faculty of Dentistry, Mansoura University, Mansoura, Egypt. Teeth were extracted for reasons unrelated to the study, including badly decayed, non-restorable molars or over-retained molars where orthodontic consultation recommended extraction.

Inclusion criteria were mandibular second primary molars with complete root length and no or minimal physiologic root resorption with at least 7 mm of root length present according to a previous study [13]. Only teeth with a single distal canal were included, as confirmed by preliminary periapical radiographs.

Exclusion criteria included internal or pathological root resorption, canal calcification, root perforation, or previous endodontic treatment.

### Sample size calculation

Sample size was estimated based on the study by Fumes et al. [10], which reported a standard deviation of 1.4 mm for canal diameter measurements in primary molars. Using the formula for estimating a population mean with 95% confidence (n = (Z × SD / E) ²), with Z = 1.96 and a desired precision of ± 0.3 mm, a minimum of 84 canals was required. As each mandibular second primary molar typically contains three canals, this corresponded to approximately 28 teeth. To compensate for possible specimen loss and exclusions, the sample size was increased to 50 teeth (150 canals). Therefore, the sample size calculation was primarily intended to ensure adequate precision for descriptive estimation of apical anatomical parameters, rather than to power formal between-canal comparisons.

### Sample preparation

Following extraction, teeth were stored in 10% formalin for two weeks and then rinsed thoroughly under running water. External debris and soft tissue remnants were removed using dental scalers. Teeth were subsequently stored in 0.1% thymol solution at 4 °C until scanning.

### Micro-CT scanning

Each molar crown was embedded in an acrylic cylinder with the horizontally flattened occlusal surface directed towards the base of the cylinder, to standardize mounting and orientation during scanning. The flat base of the acrylic cylinder was used as the mounting reference plane and was positioned parallel to the horizontal plane of the scanner turntable. Each specimen was then stabilized on the turntable using a putty rubber base material. Specimen alignment was verified using the preliminary two-dimensional projection image obtained with the scanner control software before the full micro-CT scan. On this projection image, the reference axis of each molar was defined as the root long axis, represented on the proximal aspect by a line extending from the root apex to the cementoenamel junction at the midpoint buccolingually. During mounting, this root long axis was aligned parallel to the vertical rotation axis of the scanner. Minor adjustments were made until the root axis appeared vertical, and no obvious tilting was evident. All specimens were scanned using a desktop micro-CT scanner (MicroCT SKYscan 1172; Bruker, Belgium) at an isotropic resolution of 15.24 μm, with the following parameters: 92 kV, 91 µA, 8 W, 1 mm aluminum filter, exposure time 5.9 ms, and magnification 17.24×. Samples were scanned over 180° with a rotation step of 0.45°.

### Image reconstruction and evaluation

**Fig. 1:**
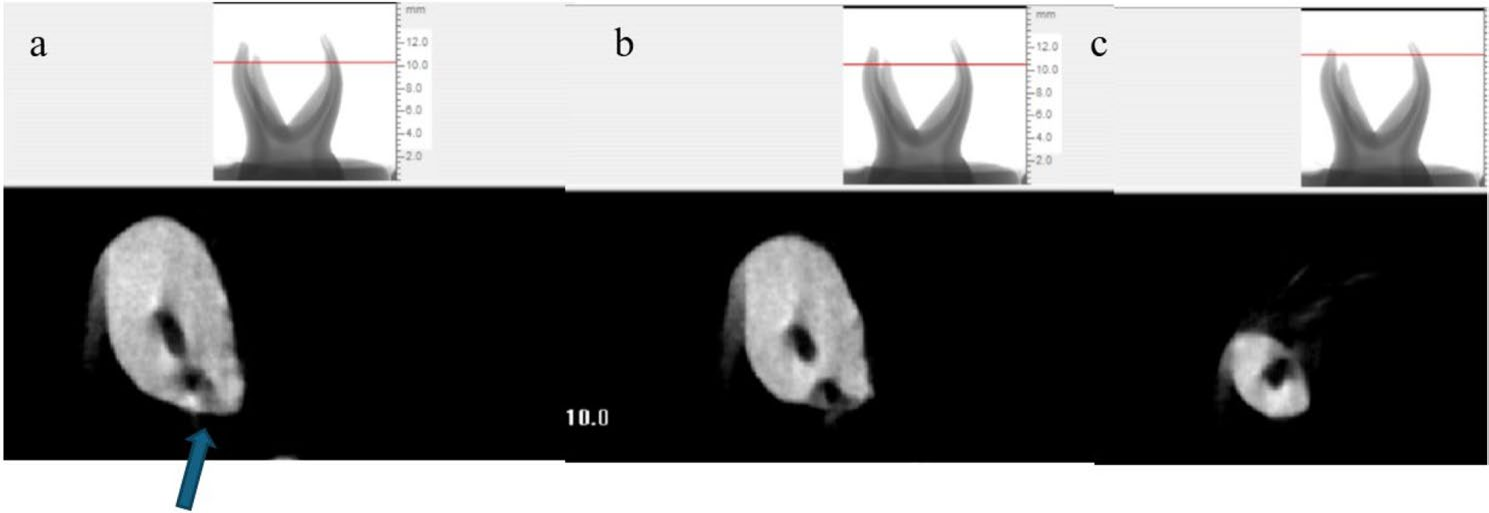
Micro-CT cross-sectional images of the mesiobuccul root apical region showing from coronal to apical direction: **a** Presence of a lateral canal (arrow) leaving the main canal. **b** The lateral canal exiting early and forming an accessory foramen. **c** The main canal exiting at the apical foramen

**Fig. 2:**
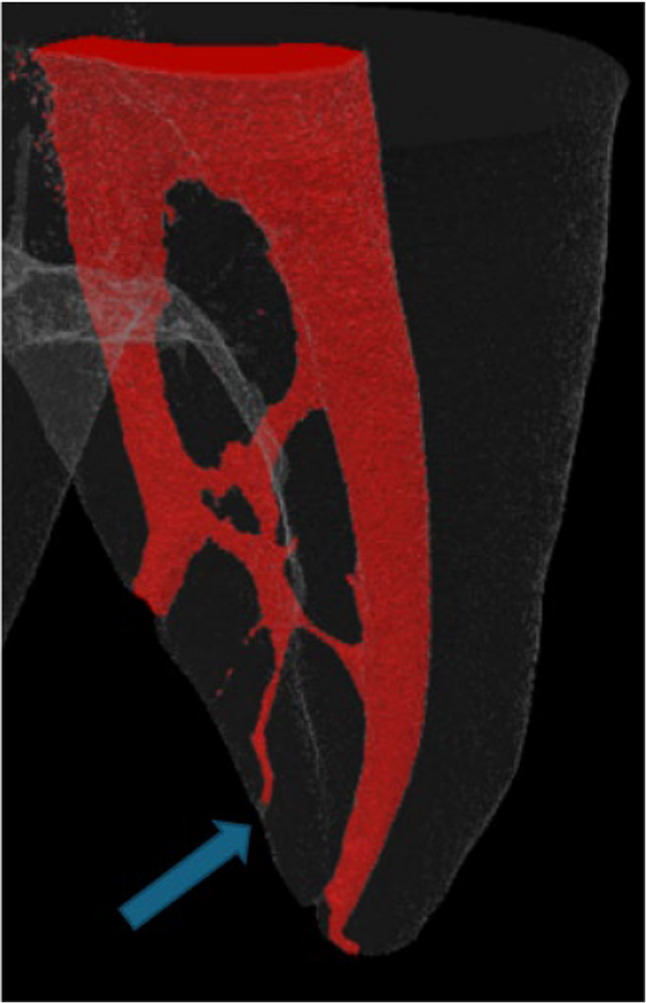
A micro-CT 3D rendered model of the mesial root of a mandibular second primary molar showing accessory canal and foramen (arrow) located midway between the main mesiobuccal and mesiolingual canals

**Fig. 3:**
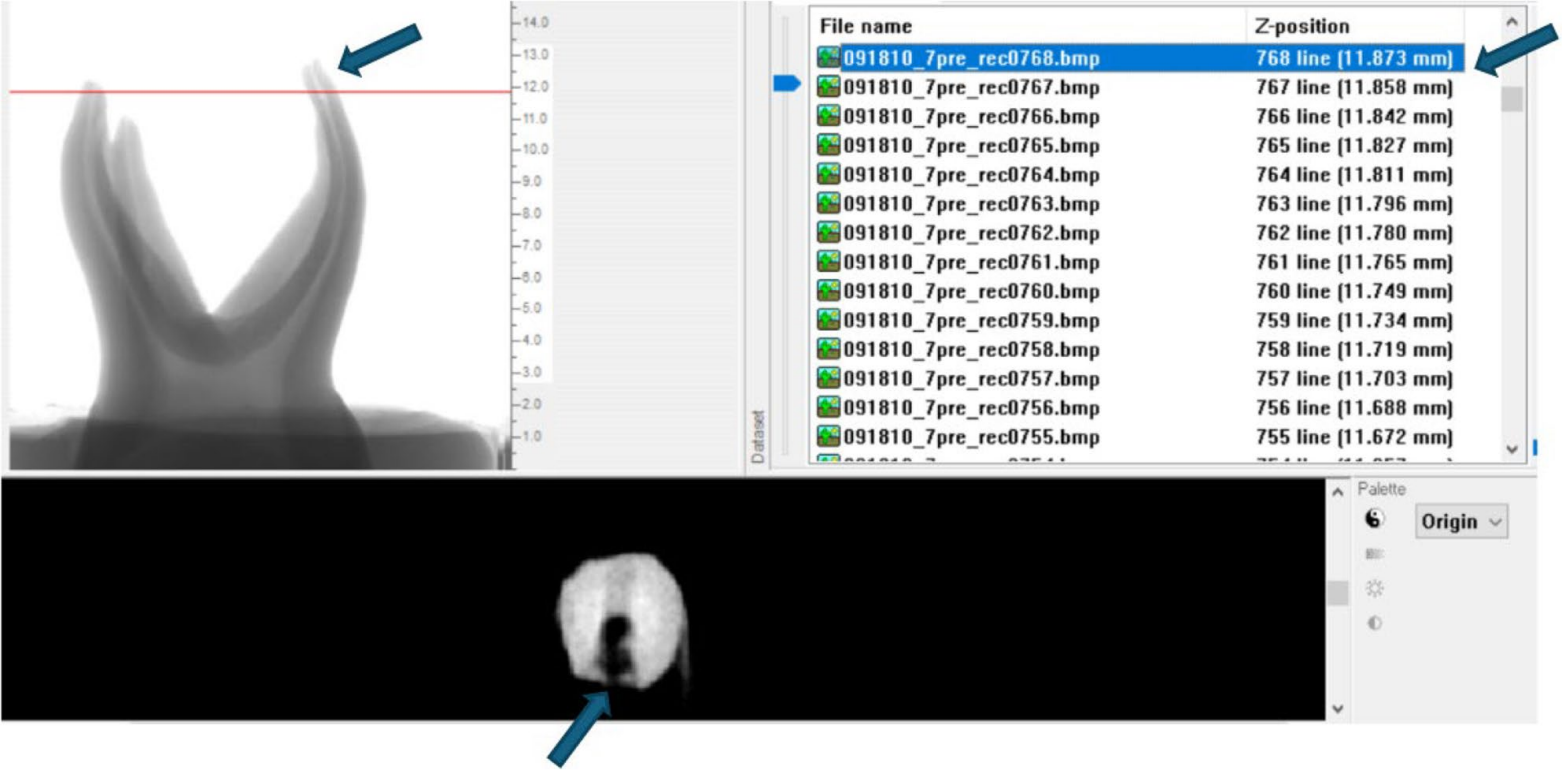
A micro-CT image of the distal root showing how the level of the apical foramen (arrow) was determined on the cross-sectional slice with its corresponding Z-position value

The acquired images (TIFF format) were reconstructed using NRecon software (version 1.6.3.2; Bruker-microCT, Kontich, Belgium). The reconstructed image stacks were visualized and analyzed using CTAn software (version 1.10.1.0; Bruker-microCT). All measurements were performed by a calibrated examiner. To assess intra-examiner reliability, measurements were repeated after a 2-week interval on the same dataset. Reliability was evaluated using the intraclass correlation coefficient (ICC), which demonstrated excellent agreement (ICC = 0.86). The apical foramen was defined as the most apically located main canal portal of exit while lateral canals or accessory foramina were not considered when present (Figs. 1 and 2). The level of the apical foramen was identified on the horizontal plane perpendicular to the long axis of the root using the two-dimensional (2D) raw image stack in CTAn program. For each canal, the image stack was examined in the Z-direction, starting from the canal orifice coronally and progressing apically along the entire canal length until the main canal lumen communicated apically with the external root surface through the main portal of exit (apical foramen). The corresponding cross-sectional slice was defined as the apical foramen level and was used as the landmark for subsequent measurements (Fig. 3).

**Fig. 4:**
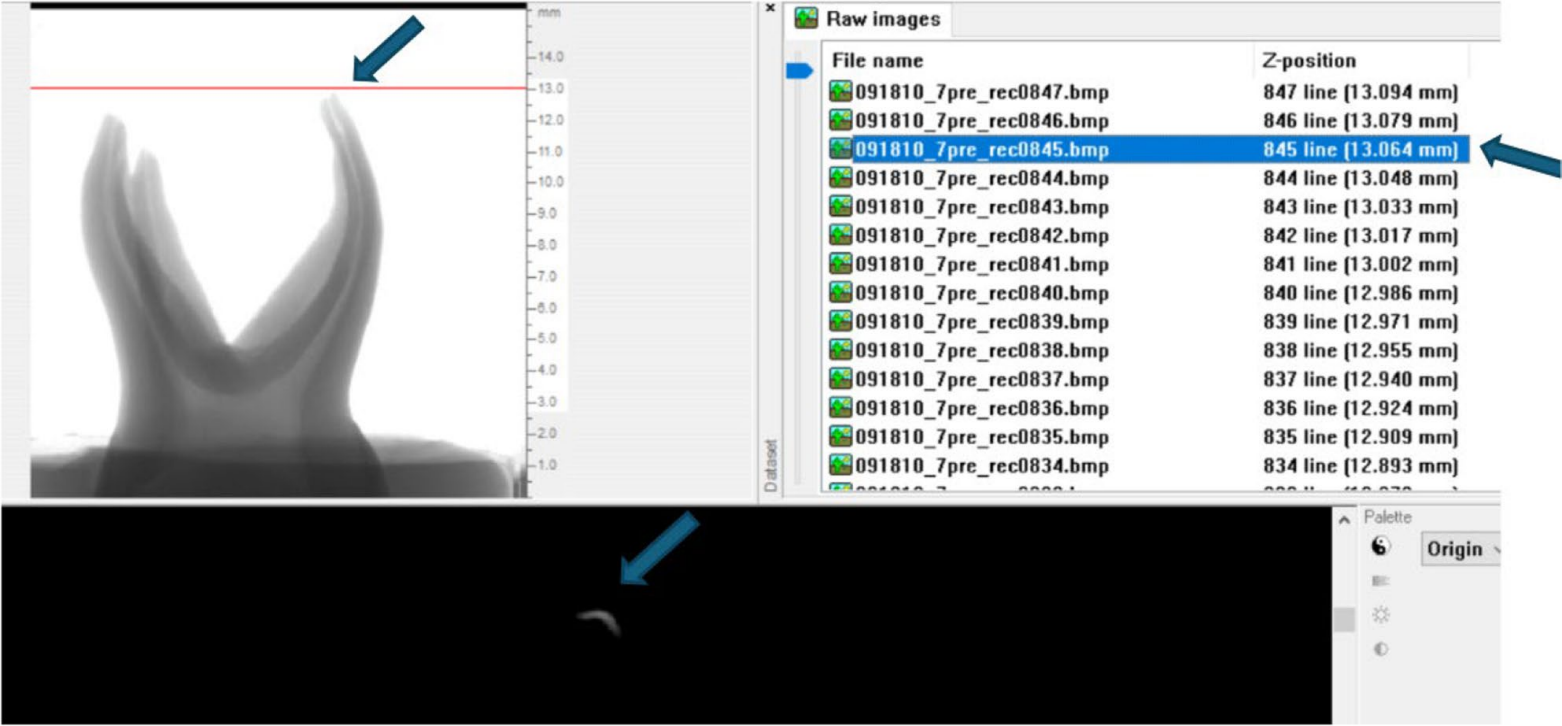
A micro-CT image of the distal root showing how the level of the anatomic apex (arrow) was determined on the cross-sectional slice with its corresponding Z-position value

The root apex was defined as the most apical part of the root identifiable on the raw image stack in CTAn (Fig. 4). The apex-to-foramen distance was calculated by subtracting the Z-position millimeter value of the slice representing the apical foramen level from the Z-position value of the slice representing the root apex. Because each slice had a defined spatial position within the specimen, this difference was recorded as the apex-to-foramen distance.

**Fig. 5:**
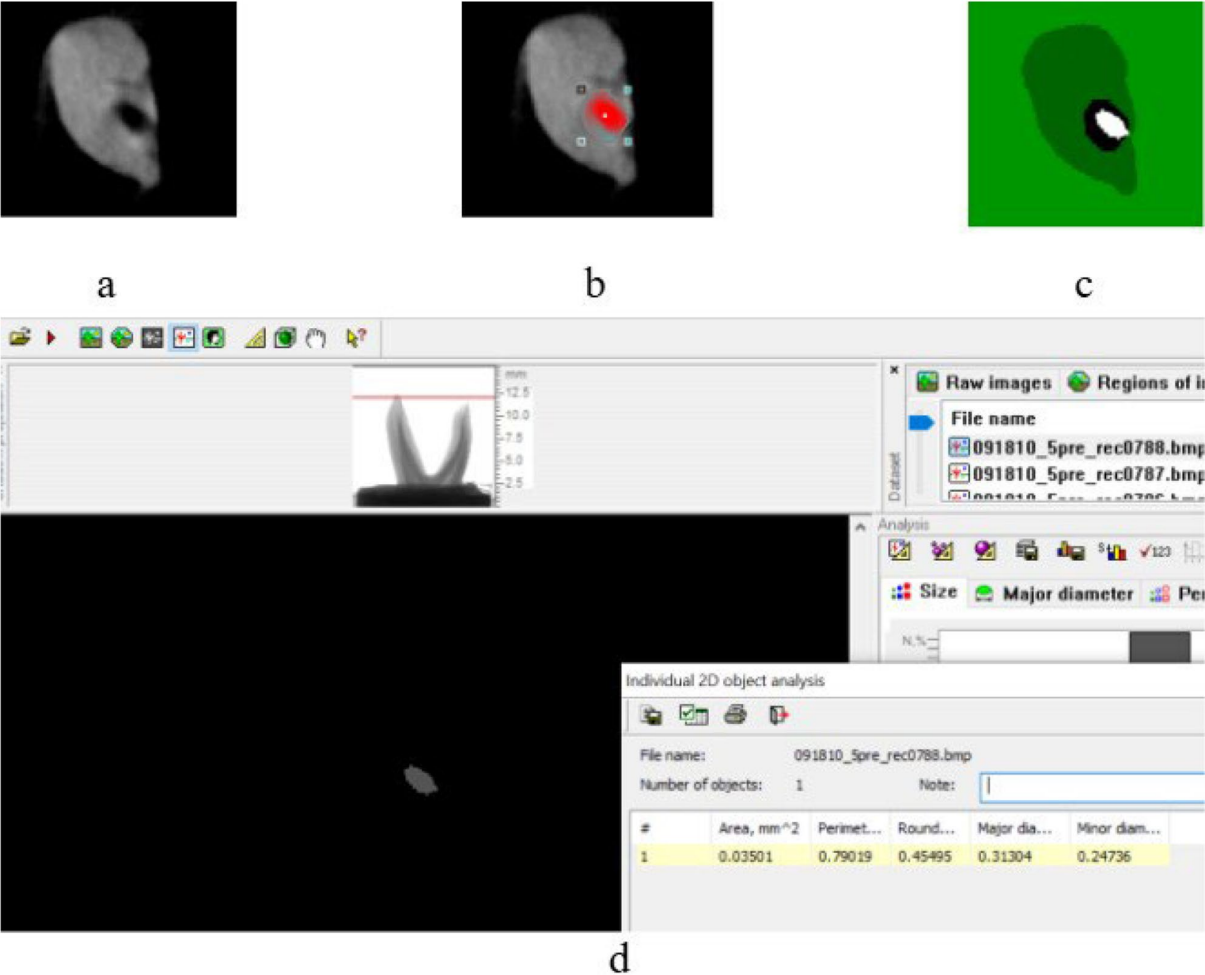
View of a micro-CT dataset slice of the mesiobuccal canal cross section at the apical foramen level; (**a**) (raw image), (**b**) (region of interest defined), (**c**) (canal lumen binarized), (**d**) (Individual 2D analysis performed).

On the raw image slice of the canal cross section at the apical foramen level, a region of interest was drawn to include the canal lumen. The binarized cross-sectional object representing the canal lumen was then analyzed two-dimensionally using the individual 2D object analysis function in CTAn to obtain the major diameter and roundness values (Fig. 5). The major diameter was defined as the maximum distance between the two most distant pixels within the object. Roundness values range from 0 to 1, with 1 indicating a perfect circle [10].

### Statistical analysis

Statistical analysis was performed using IBM SPSS Statistics (version 20.0; IBM Corp., Armonk, NY, USA). The Shapiro–Wilk test was used to verify the normality of the data distribution. To ensure the appropriateness of the parametric tests, Levene’s test for homogeneity of variances was applied. Quantitative variables were expressed as mean ± standard deviation (SD), median [interquartile range], and range (minimum–maximum).

Differences among the three canal types (distal, mesiobuccal, and mesiolingual) were evaluated using one-way analysis of variance (ANOVA). For parameters demonstrating significant differences, Tukey’s HSD post-hoc test was utilized for pairwise comparisons. Intra-examiner reliability was assessed using the intraclass correlation coefficient (ICC) based on a two-way mixed-effects model. The significance level for all tests was set at α = 0.05.

## Results

A total of 150 root canals (50 distal, 50 mesiobuccal, and 50 mesiolingual) from 50 mandibular second primary molars were analyzed. Descriptive statistics of apical anatomical parameters are presented in Tables 1 and 2.

**Table 1:** Apical anatomical parameters (overall sample)

Variable	*n*	Mean ± SD	Median [IQR]	Min–Max
Apex-to-foramen distance (mm)	150	0.57 ± 0.44	0.40 [0.60]	0.10–1.60
Apical canal diameter (mm)	150	0.63 ± 0.24	0.70 [0.40]	0.20–1.00
Roundness	150	0.48 ± 0.17	0.50 [0.20]	0.10–0.80

Values are presented as mean ± SD, median [IQR], and range (min–max)

**Table 2:** Apical anatomical parameters according to canal type

Parameter	Canal type	n	Mean ± SD	Median [IQR]	Min–Max	ANOVA p-value
Apex-to-foramen distance (mm)	Distal	50	0.64 ± 0.42	0.70 [0.50]	0.10–1.30	0.040
	Mesiobuccal	50	0.44 ± 0.15	0.40 [0.20]	0.30–0.70	
	Mesiolingual	50	0.62 ± 0.59	0.20 [0.80]	0.10–1.60	
Apical canal diameter (mm)	Distalᵃ	50	0.74 ± 0.30	0.80 [0.30]	0.20–1.00	< 0.001
	Mesiobuccalᵇ	50	0.56 ± 0.22	0.69 [0.40]	0.30–0.80	
	Mesiolingualᵇ	50	0.59 ± 0.15	0.60 [0.25]	0.40–0.80	
Roundness	Distal	50	0.45 ± 0.25	0.40 [0.36]	0.10–0.80	0.079
	Mesiobuccal	50	0.52 ± 0.08	0.50 [0.10]	0.40–0.60	
	Mesiolingual	50	0.46 ± 0.10	0.50 [0.10]	0.30–0.60	

Values are presented as mean ± standard deviation, median [interquartile range], and range. *p*-values were calculated using one-way ANOVA. Different superscript letters indicate statistically significant pairwise differences according to Tukey HSD post-hoc test (*p* < 0.05). For distance from apex, the overall ANOVA was significant, but Tukey post-hoc comparisons showed no significant pairwise differences

Overall, the apical foramen was located at a mean distance of 0.57 ± 0.44 mm from the apex (median 0.40 mm; IQR 0.60; range 0.10–1.60 mm). The mean apical canal diameter at the foramen level was 0.63 ± 0.24 mm (median 0.70 mm; IQR 0.40; range 0.20–1.00 mm), and the mean roundness was 0.48 ± 0.17 (median 0.50; IQR 0.20; range 0.10–0.80).

**Fig. 6:**
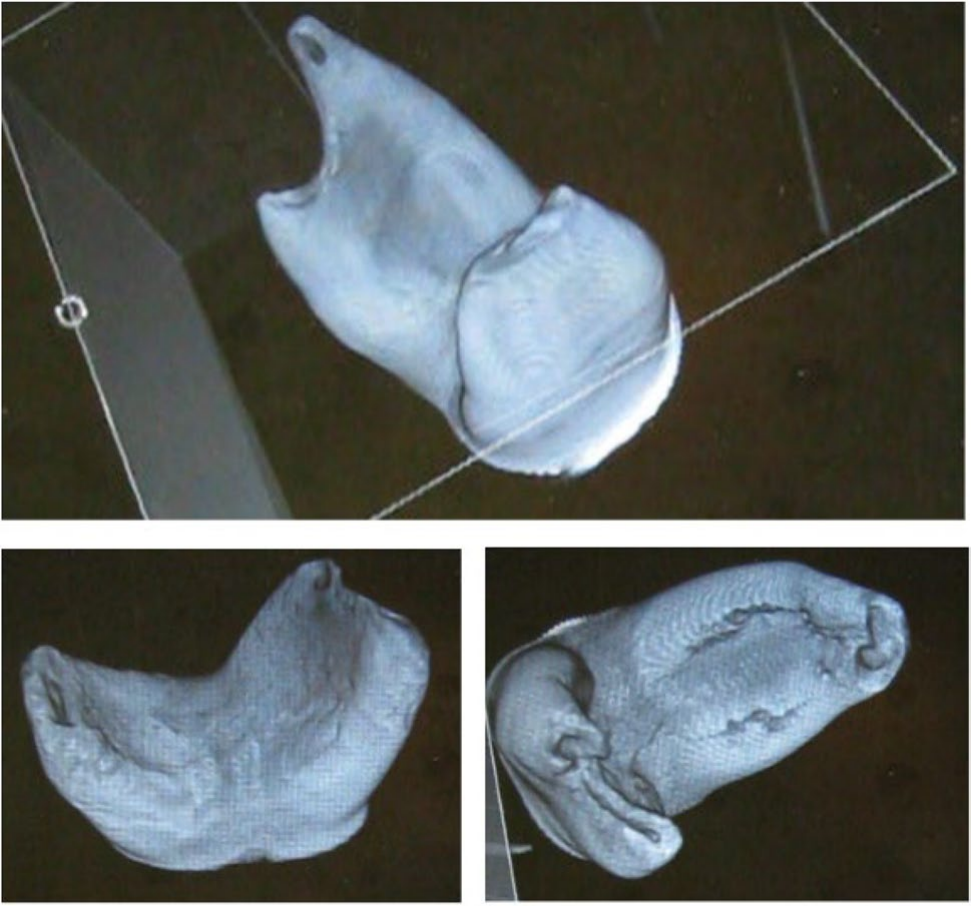
Micro-CT 3D rendered models of mandibular second primary molars showing a lack of coincidence between the apical foramen and the anatomic apex

The apical foramen did not coincide with the anatomic apex in any canal (Fig. 6). The overall ANOVA showed a statistically significant difference in apex-to-foramen distance among canal types (*p* = 0.040). However, Tukey post-hoc comparisons did not identify statistically significant pairwise differences between individual canal types; therefore, no specific canal-type difference could be confirmed.

**Fig. 7:**
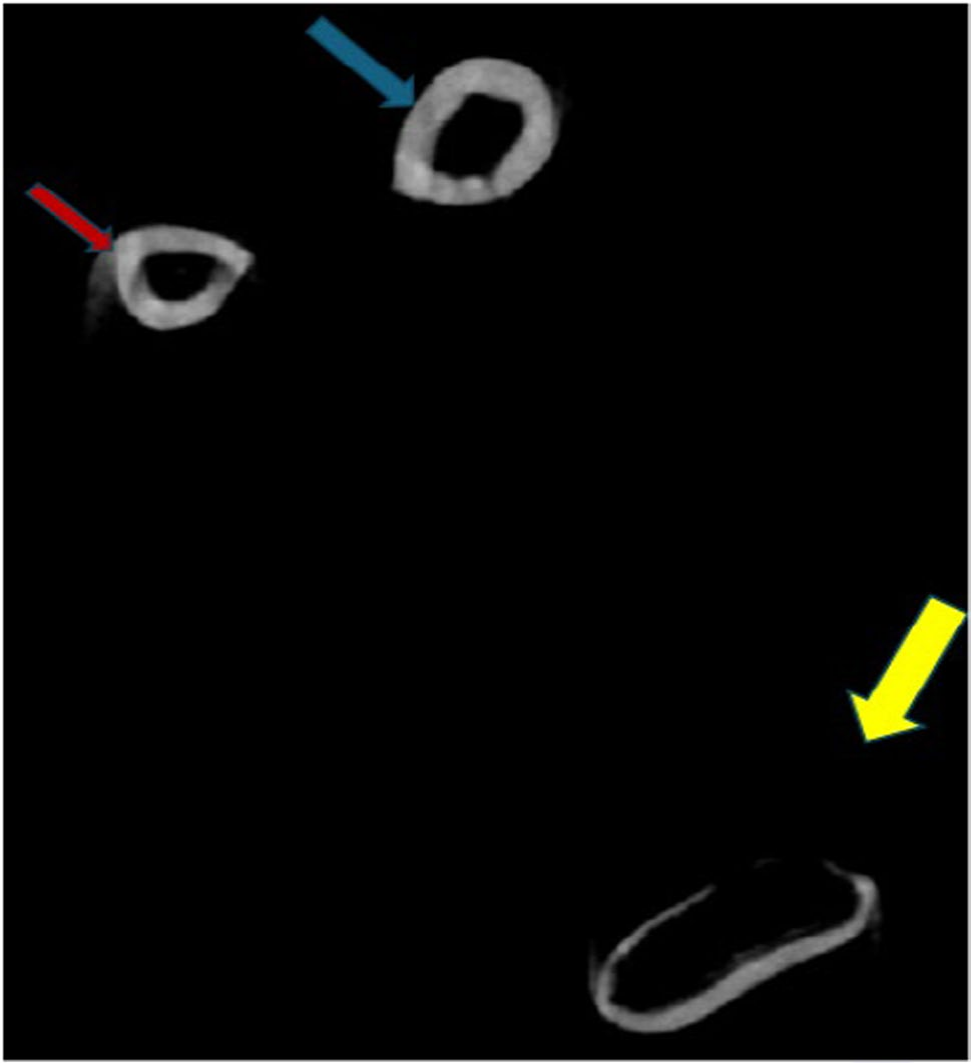
A micro-CT cross-sectional image of mandibular second primary molar roots at the apical foramen level showing the variable diameter of root canals. Distal (yellow arrow), mesiobuccal (blue arrow), and mesiolingual (red arrow)

Apical canal diameter showed a highly significant difference among canal types (ANOVA, *p* < 0.001). Distal canals exhibited significantly larger diameters than both mesiobuccal and mesiolingual canals (*p* < 0.05), whereas the difference between mesiobuccal and mesiolingual canals was not significant (Fig. 7). Roundness did not differ significantly among canal types (ANOVA, *p* = 0.079).

## Discussion

Primary molars exhibit complex and highly variable root canal anatomy, which may pose significant challenges during endodontic treatment [14]. Understanding this anatomy is essential for selecting appropriate instrumentation strategies to achieve effective cleaning and shaping while minimizing procedural errors [10].

In the present study, the apical anatomy of mandibular second primary molar was investigated. This tooth is strategically important in young children, particularly before eruption of the first permanent molar, as it contributes to preservation of arch length and occlusal stability. Moreover, when indicated, successful endodontic management of this tooth may support long-term retention in cases of congenital absence of permanent successors [15].

The molars included in this study were selected with complete root length and minimal root resorption, as physiologic resorption alters apical morphology and changes the spatial relationship between the apical foramen and the anatomic apex due to the asymmetric resorption pattern along root surfaces [16]. To ensure complete root length and absence of root resorption exceeding one third of the root length, the root was measured from the cemento-enamel junction to the anatomic apex and had to be at least 7 mm according to the average root length for primary mandibular molars in Egyptian children as shown in the study by El-Messiry et al. [13]. Using Micro-CT enabled accurate quantitative assessment of apical anatomical variables, including the distance from the apical foramen to the apex, the major diameter and roundness of the root canal at the foramen level. This level was selected because it represented a well-defined and reproducible apical landmark corresponding to the most apical level of the main canal immediately before its communication with the external root surface through the apical foramen. This landmark could be identified on cross-sectional 2D root canal slices using CTAn software. It was therefore considered the most appropriate anatomical level for conducting the measurements in the present study. Clinically, the apical foramen is also relevant because it can be detected using EALs. Ideally, endodontic instrumentation and obturation should be confined within the root canal lumen and terminate at the apical constriction. However, unlike permanent teeth, primary teeth may lack a clearly defined apical constriction. In addition, physiological root resorption, even at its early stages, may continuously alter the apical region, resulting in a wider and more variable apical foramen [16].

The findings demonstrated that the apical foramen did not coincide with the apex in any canal, with a distance ranging from 0.1 to 1.6 mm and a mean of 0.57 mm. Clinically, this supports the importance of (EALs), which have shown high accuracy in working length determination [17]. Reliance on radiographs alone may be misleading and may increase the risk of over-instrumentation, which is particularly undesirable in primary molars due to the proximity of the developing permanent tooth germ. This concern becomes even more critical in teeth with root resorption, where the foramen usually shifts coronally due to unequal resorption patterns [18].

In this study, the relatively small range reported for the distance from the apical foramen to the apex may be likely due to the consistent inclusion of molars with no or minimal resorption. In contrast, Alafandy et al. [16] reported a wider range for the distance from the foramen to the apex (0–6 mm) with a mean of 1 mm, which may be attributed to inclusion of both maxillary and mandibular molars with varying degrees of root resorption.

Regarding apical canal diameter, the distal canals exhibited the largest mean diameter (0.74 ± 0.30 mm), whereas mesiobuccal canals showed the smallest (0.56 ± 0.22 mm) with mesiolingual canals demonstrating intermediate values (0.59 ± 0.15 mm). This is consistent with the results of Zoremchhingi et al. [9] who demonstrated that distal canals of second primary molars had the maximum diameter at cervical, middle, and apical levels, while the mesiolingual canal had the minimum diameter. Gaurav et al. [19] also reported that the distal canal in mandibular primary molars had the maximum diameter, although in their study the canal diameter was measured only at the cervical and middle thirds of the root. Similarly, El Hachem et al. [20] reported that the buccolingual diameter was greater in coronal, middle, and apical levels, in the distal root that only had one root canal. In clinical endodontics, apical canal width is a key determinant in shaping and disinfection as adequate apical enlarging allows maximum mechanical debridement and deeper penetration of the irrigant into the canal for optimal disinfection which may improve treatment outcome in teeth with periapical lesions [21]. However, the apical preparation size or working width remains under-investigated and has been described as the “forgotten dimension” in endodontics, and the question of how large is large enough still needs to be addressed [22]. Traditional pediatric endodontic recommendations include enlarging the canals several sizes beyond the first apically binding file to a minimum final size of 30–35 [23, 24] or enlarging up to size 40 or 50 [25]. However, these preparation sizes are not supported by evidence and may not be applicable in all cases [22]. Based on the present study findings, instrumentation sizes would theoretically correspond approximately to size 80 file to fully prepare the circumference of the apical area in distal canals and size 60 for both mesiobuccal and mesiolingual canals. This is consistent with Fumes et al. [10], who suggested that debridement in primary molars may require instruments up to size 100.

Nevertheless, such large apical enlargement is clinically unrealistic and may increase the risk of excessive dentin removal and weakening of root structure, particularly when using instruments with tapers greater than 0.02. Therefore, careful instrument selection and adjunctive disinfection methods, such as passive ultrasonic irrigation or negative apical pressure systems, may be necessary to improve apical cleaning. The present results highlight the essential role of chemical disinfection and the antibacterial and sealing ability of obturation materials in achieving three-dimensional disinfection and sealing of the canal system.

The results may also support reconsideration of current instrumentation approaches in primary molars. A hybrid instrumentation strategy may be beneficial, such as using larger-taper rotary instruments only for coronal flaring and minimal-taper instruments (0.02) with considerable adequate size for safely enlarging the apical preparation. Instruments designed to adapt to irregular canal anatomy, such as the self-adjusting file (SAF), have been proposed to improve shaping and cleaning [10, 26, 27]. However, to date no instrumentation system can completely clean or disinfect the apical region, and an effective irrigation protocol remains essential [28, 29].

Regarding apical canal shape, roundness index values did not differ significantly among canal types, indicating a comparable cross-sectional configuration. Values were generally less than 1, suggesting an oval morphology, which may contribute to incomplete apical preparation when round instruments are used. Contrary to the present findings, Kurthukoti et al. [30] in their computed topography (CT) study of mandibular second primary molars showed that all the canals demonstrated a round cross section at the apical level. However, their methodology relied on a lower resolution imaging technique (CT), and the canal cross sections were evaluated qualitatively.

The present study primarily aimed to investigate and describe selected apical anatomical features of mandibular second primary molars. Comparisons among different canal types were considered a secondary objective, as variations in canal configuration and apical diameter may have clinical relevance by influencing the selection of instrument size and taper to better match the anatomical characteristics of each canal. This is clinically important because the use of a single-file preparation approach for all canals within the same primary molar may not adequately account for anatomical differences among canals.

To date, no standardized protocol exists for root canal preparation in primary teeth [31]. Future research should focus on establishing evidence-based instrumentation strategies tailored to the unique morphology of primary molar canals, particularly in the apical region. The apical region is particularly important in cases of necrotic or infected teeth as this difficult to access part may harbor a considerable number of microbial species [32] that have better accessibility to periapical tissue and can form microbial biofilm extending to external root surface (extra radicular infection) in cases of longstanding chronic apical periodontitis presenting with periapical radiolucency [33]. This biofilm could be responsible for persistent infection and treatment failure [34]. Therefore, it may be necessary for infected teeth that the whole root canal should be disinfected to the level of the apical foramen [35]. It is therefore of utmost importance to select suitable instrumentation and disinfection techniques in each case for efficient biomechanical preparation without endangering the fragile root structure or harming the periapical tissue and the developing successors.

Significant geographical variations in the root canal anatomy of primary molars have been confirmed by Swaminathan et al. [36] in their recent systematic review, emphasizing the need for population‑specific endodontic strategies. They also highlighted the importance of population‑specific research to guide clinical decision‑making as regional variations and geographical diversity in root canal morphology cannot be ignored with the high prevalence of complex root canal configurations demonstrated in certain populations. In the light of the few studies existing in the literature targeting specific populations relative to primary teeth morphology, the present study is the first one to investigate the apical root canal anatomy of primary molars in Egyptian children. However, it can be considered a preliminary investigation and should be followed by more comprehensive research involving various morphological variables for different teeth in the primary dentition.

### Limitations

This study has limitations. First, it was conducted in vitro and included a relatively limited sample size due to difficulties in collecting specimens that met the inclusion criteria. Second, only mandibular second primary molars were evaluated; therefore, the findings may not be generalizable to other primary molars.

## Conclusion

Within the limitations of this in vitro micro-CT study:

1. The apical foramen was not coincident with the anatomic apex in the mandibular second primary molar across all root canal types.
2. The apical canal demonstrated considerable dimensional variability among canals, which may necessitate greater attention to instrumentation strategies and apical preparation protocols in pediatric endodontics.

## Data Availability

The datasets generated and analyzed during the current study are available from the corresponding author upon reasonable request.
